# Dual-balloon assisted super-selective embolisation of high flow arterial venous fistula within a transplant kidney

**DOI:** 10.1186/s42155-018-0029-x

**Published:** 2018-10-30

**Authors:** Sara ffrench-Constant, Nisal Weerakoon, Rahul Amin, Luke Dixon, David Taube, Mohamad Hamady

**Affiliations:** 1 Imperial College Healthcare Trust, London, UK; 2 Imperial College London, London, UK; 3 Imperial College Health Trust, London, UK

**Keywords:** Embolisation, Balloon assisted embolisation, Iatrogenic fistula, Renal transplant, High flow arteriovenous fistula

## Abstract

**Background:**

In this case report, we describe a novel application of the technique of ‘dual-balloon assisted’ cannulation and embolisation of a high flow arterial venous fistula (AVF) in transplant kidney, where attempts at standard and previously described embolisation techniques were proving difficult to achieve.

**Case presentation:**

Seventy year old gentleman with renal transplant presenting with high output cardiac failure and deteriorating renal function. Angiography demonstrated high flow traumatic AV fistula within transplanted kidney, secondary to multiple biopsies. Attempts at guidewire and guiding sheath placement and stability for occlusion plug deployment were failing due to combination of very high back-flow pressures within the AVF and challenging vascular anatomy; with an aneurysmal, tortuous iliac artery as well as intra renal transplanted vessels. A combination of angioplasty and remodeling aortic balloons in the transplant artery and the host external iliac vein respectively, facilitated stabilization of guiding sheath and hence controlled delivery of an occlusion plug from the venous side of the fistula. The fistula was successfully embolised, leading to complete resolution of patient symptoms and improvement of renal function beyond his previous baseline.

**Conclusions:**

Percutaneous embolisation is an established technique to treat iatrogenic AVF in transplant kidneys. High flow pressure through an AVF, as demonstrated in this case, can cause difficulty and raise safety issues in accessing and embolising the AVF using previously described techniques. This case report describes an effective and novel application of the technique of using a second balloon in the host common iliac vein to; lower flow pressure, stabilise the guidewires during plug deployment and prevent displacement of wires and/or plug into the common iliac vein.

## Background

An arteriovenous malformation is an abnormal communication between arterial and venous systems. An acquired communication, as opposed to a developmental one, is an arteriovenous fistula (AVF) (Eibl et al. 2009). The incidence of intrarenal AVMs is fairly low, estimated to be around 0.3% in native kidneys. However, the incidence of AVFs is much higher, especially in transplant kidneys - closer to 6–8% (Vij et al. 2011). AVFs have long been a recognized complication post renal transplant biopsy (Merkus et al. 1993; Diaz-Buxo et al. 1974). Whilst these are often quiescent, if left untreated, they can lead to deterioration in renal function and high out-flow cardiac failure (Maldonado et al. 1964). Once symptomatic, an AVF requires treatment – of which embolization is the favoured technique. This can be achieved by a number of minimally invasive transcatheter methods, including occlusion plug embolization (Rajesh et al. 2015) and coil embolization (Sundarakumar et al. 2013). High flow in the fistula usually imposes a significant safety challenge during intervention. This case describes a novel application of the technique of dual balloon occlusions facilitating plug embolisation from the venous side of the fistula. Ethical approval was obtained according to our institutional guidelines.

## Case presentation

We present a case of a 70-year old Caucasian gentleman who underwent a living donor renal transplant 10 years ago following a diagnosis of glomerulonephritis aged 34. More recently, he had developed acute heart failure manifested by bilateral leg oedema, facial oedema and difficulty in breathing together with severe and difficult to control hypertension with wide pulse pressures. Biochemistry at the time of presentation revealed Cr 190 mmol/L and eGFR 27–32.

**Fig. 1:**
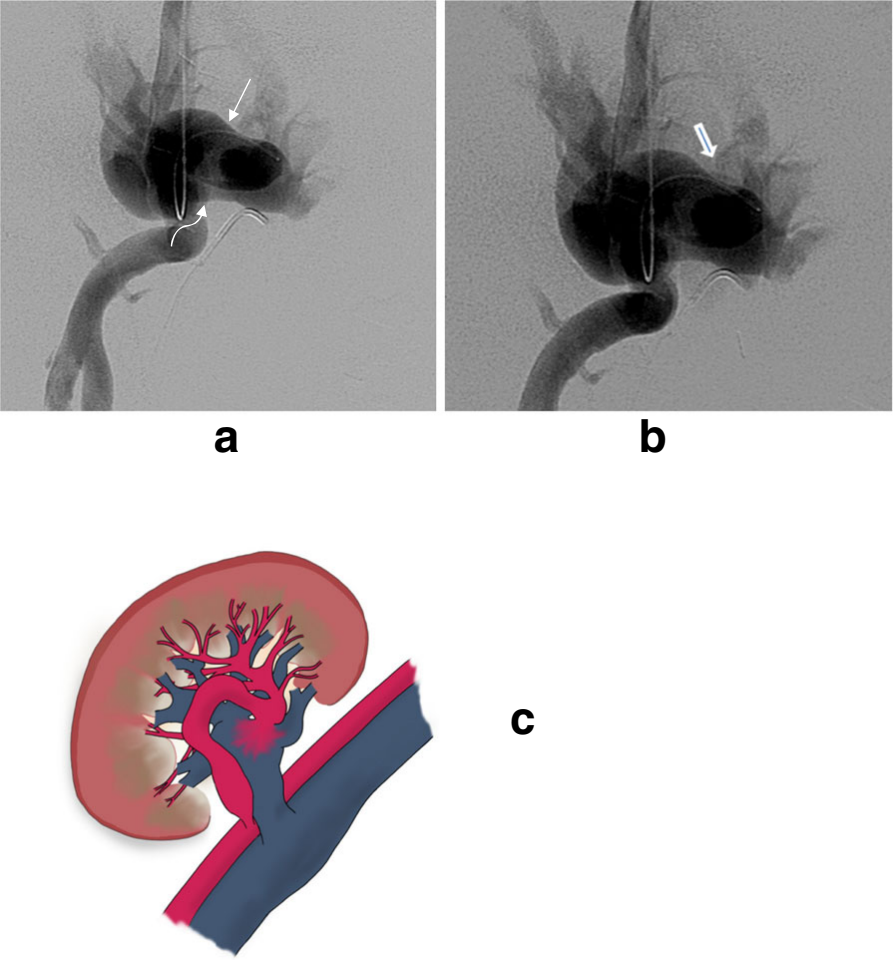
Angiographic and schematic demonstration of high flow arterio-venous fistula within the transplanted kidney. **a** Aneurysmal intra renal artery (white arrow) and relative narrowing at the AVF connection (curved arrow) demonstrated on pre-procedure angiography. **b** Left lateral oblique view shows early draining aneurysmal renal vein (solid arrow). **c** Schematic diagram demonstrating the high flow AVF within the transplant kidney and highlighting the tortuous vessel anatomy

**Fig. 2:**
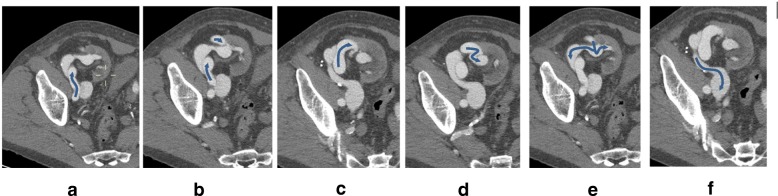
Series of CTA images demonstrating high flow arterio-venous fistula within the transplanted kidney. Block arrow demonstrating route of blood from transplant artery (images **a-d**), the AV fistula (image **e**) and the early filling of the transplant renal vein (image **f**)

The patient had past history of several transplant biopsies performed to investigate repeated rise in serum creatinine level over the last 10 years. Clinically, there was audible bruit over the right side of the lower abdomen. Ultrasound, CT angiography scan and subsequently catheter angiography demonstrated very high flow arterial venous fistula within the transplanted organ (Fig. 1), likely to be the cause of the patients symptoms of decompensated heart failure. The main transplant artery and veins, as well as all intra renal branches were aneurysmal with extreme tortuosity, especially in the intra renal vessels. The maximum diameter of the main transplant artery was 12 mm with relative narrowing at the origin. Other salient findings were ectatic and tortuous iliac vessels (Fig. 2). Given the decompensated heart failure being caused as a result of the high flow AVF, patient planned for super-selective embolisation of the arterio-venous communication under interventional radiology.

Informed consent was obtained and patient readmitted for elective super selective renal transplant embolization, to be performed under general anaesthesia in order to control the patient’s haemodynamic status. Given the knowledge of high flow through the AVM, plans were made to make both arterial and venous punctures. The contralateral left common femoral artery was punctured under ultrasound guidance and 6F sheath was inserted and positioned in the right common iliac artery. Through the sheath, the transplant artery was selected and 6mmx40mm angioplasty balloon was positioned and inflated at the origin of the transplant artery to reduce inflow and hence pressure into the AVM. The site of the AVM was approached via the venous side.

Since the arterial side has a tight, almost 360°, backward bend to AV communication site and the venous channel has shorter and straighter path, a 6F sheath was inserted into the right common femoral vein, aiming to deliver the embolic device. Multiple attempts at cannulating the fistula from the venous side failed due to the predicted factors previously described. The tortuosity of the vessels led to difficult in reaching the exact site of the AVM, whilst the high flow from the arterial side continually forced the guide wires and catheters out of the transplant vein and back up into the common iliac vein as the inflated balloon at the origin of the artery failed to reduce the flow.

**Fig. 3:**
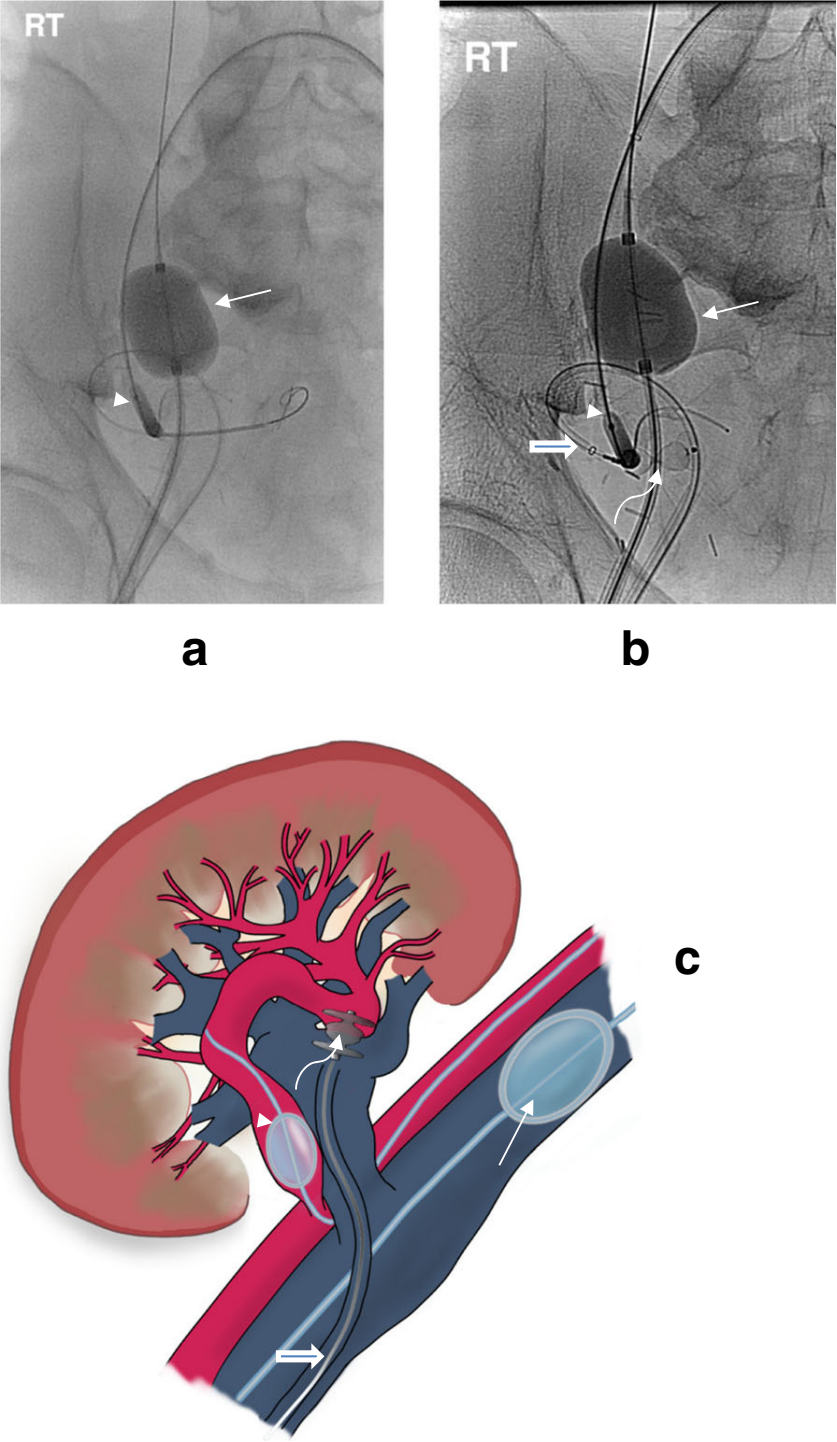
Super-selective embolisation of the AVF. **a** and **b**Transient balloon occlusion of the artery (arrow head) and common iliac vein (white arrow) to allow advancement of the sheath (solid arrow) and deployment of Amplatzer plug (curved arrow) across AVF. **c** Schematic diagram demonstrating the above embolisation technique

**Fig. 4:**
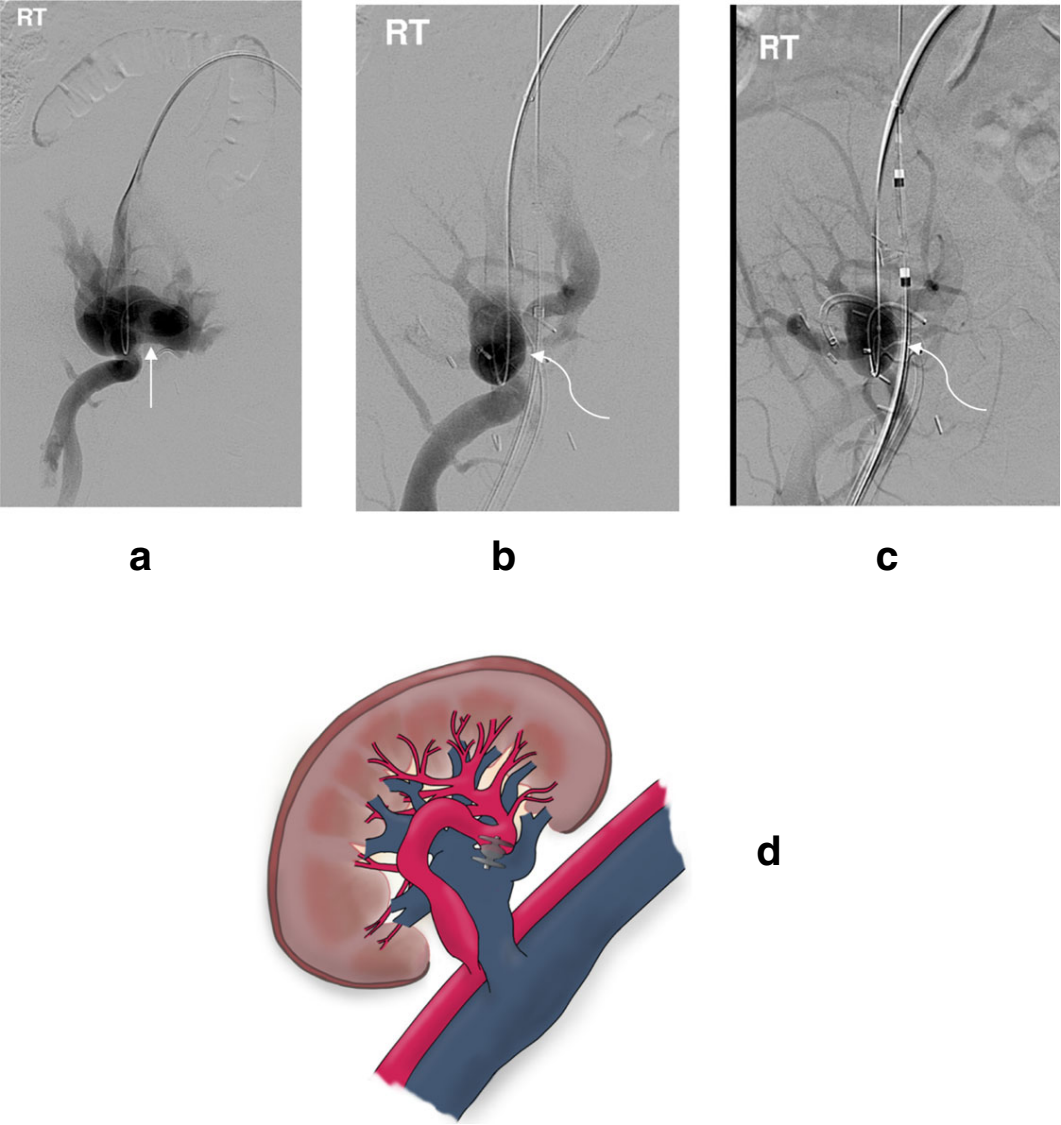
Angiographic demonstration of successful embolisation of AVF. The AVF (white arrow) demonstrated pre (**a**) and post (**b** & **c**) successful Amplatzer plug (curved arrow) occlusion, with no filling of the fistula. **d** Schematic diagram confirming Amplatzer plug occlusion of the AVF with no through flow

Therefore, an additional 12F sheath and balloon (Medtronic Reliant^M^ stent graft balloon catheter) were inserted into the venous ipsilateral side, coming from an insertion site just above the initial puncture. This balloon allowed transient occlusion of the common iliac vein (Fig. 3a), to ensure the guide wire remained in the transplant vein and not forced back into the host common iliac vein by the arterial pressure coming through the AVM. Once the wire was secured in situ, an occlusion plug (10mmx7mm) (AMPLATZER ™ Vascular Plug II, Abbott) was advanced through a 6F sheath and deployed across the fistula (Fig. 3b). This led to good embolic occlusive result with subsequent angiography showing no flow across the previous fistula (Fig. 4 – (a) pre occlusion plug and (b&c) post occlusion plug).

Following successful embolisation of the fistula, patient’s haemodynamic status remained stable. There was a transient rise in serum creatinine immediately after the procedure due to contrast nephrotoxicity from the procedure itself. In the proceeding weeks, the blood pressure decreased to around 130/85 systolic and serum creatinine improved to 138 on latest bloods. Of most importance, the patient is clinically significantly improved and has reported complete resolution of breathlessness and oedema – suggesting his symptoms were a direct result of the AVF causing high flow cardiac failure.

**Fig. 5:**
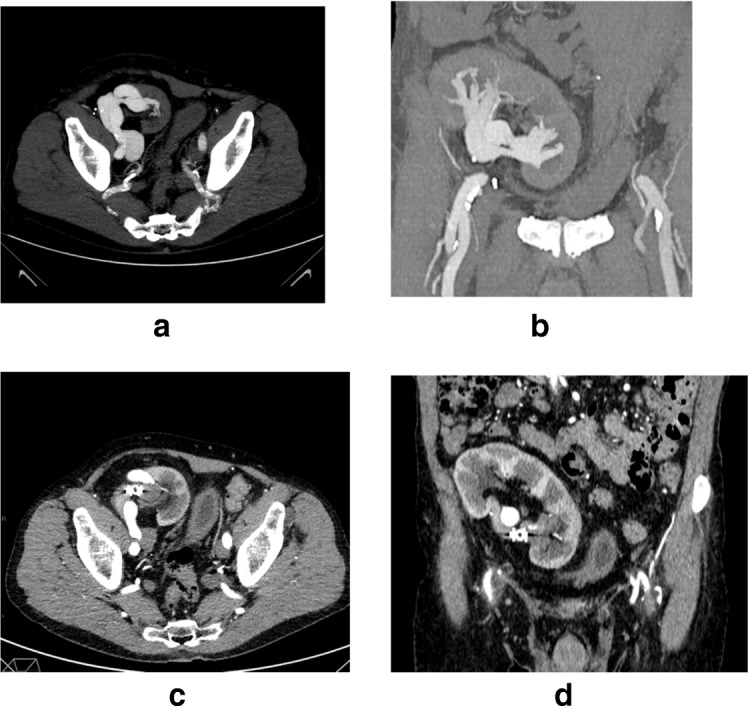
2-month follow up CT angiogram. Pre procedure axial (**a**) and coronal (**b**) and post procedure axial (**c**) and coronal (**d**) images prove successful closure of AVF with Amplazter plug and no perfusion defect

USS of the transplanted kidney performed 1 week after intervention showed good perfusion and no evidence of a residual or recurrent AV fistula. Follow up CT Angiogram performed 2 months after procedure confirmed good perfusion of transplanted kidney, stable position of the occlusion device at AVF site and resolution of the previous AVF (Fig. 5).

## Discussion

Angiographic embolization of arteriovenous fistulae is a successful treatment in patients with renal transplant AVF and a technique that has been well described in the literature. The benefit of this therapy is highlighted in a retrospective review of embolic therapy utilizing a super-selective technique. Twenty-one renal transplant patients with iatrogenic AVF’s were reviewed, with successful embolisation demonstrated in 95% of cases with no serious complications (Perini et al. 1998). Effective therapy led to eradication of main presenting symptom in 88% of patients with 58% demonstrating no detrimental effect on transplant function long-term. A similar case series followed selective embolisation of 10 renal transplant AVFs secondary to renal biopsy (Schwarz et al. 2008). These 10 cases were from an initial cohort of 2824 renal transplant biopsies, with AVFs confirmed by Doppler US in 235 of these (8.3%). On follow up, only 30 patients had persistent AVFs, with the remaining spontaneously resolving. Of the 10 selective embolisations performed on symptomatic AVFs, 9 were technically successful with no complications reported. Interestingly, improved graft function was only noted in 60%, highlighting the fact that successful embolisation does not always correspond to improved outcome. The remaining case in this review led to unsuccessful closure of the AVF and resulted in a small area of infraction in the transplant parenchyma. A final case review of 13 patients undergoing selective embolisation for symptomatic AVFs post biopsy, demonstrated successful embolisation in all patients (Maleux et al. 2003). Resolution of symptoms was seen in all 13 and improvement in graft function in 10. Only one complication was observed in this study, with thrombosis of a segmental artery, immediately treated with in situ thrombolysis and no long term ill effect. In a summary of the cases described so far, whilst renal transplant biopsies are performed commonly, the complication of a symptomatic AVF resulting from a biopsy is rare. Despite this, successful embolisation has been reported in the majority of cases, with most of these leading to improved renal transplant function and/or amelioration of the presenting symptom.

In the vast majority of the cases described in the literature, access to the AVF is gained via the arterial side and occlusion achieved with plugs or coils. Dual-balloon assisted embolisation technique has been previously described in the same and in other vascular territories (Shih et al. 2010; Barley et al. 2006). However, in our case report we embolised the fistula from the venous side while two balloons were inflated on either sides of the fistula to combine; reduction in blood flow, facilitate sheath advancement through severe tortuosity and prevent guide wire misplacement as well as occlusion plug migration. We feel this new technique of using a second balloon to stabilize the guide wires and prevent displacement into the common iliac vein can be exploited to aid access in similar cases.

## Conclusions

In conclusion, the occlusion of high flow AVF associated with extreme vessel tortuosity, vessel ectasia and short connecting channel can be achieved using dual occlusion balloons and embolisation via venous approach. The occlusion balloons will not only reduce speed of flow and minimize risk of device dislodgment but also smooth the convoluted path of guiding sheath or catheter to reach the target point.

## Abbrevations

AVF: Arterio-venous fistula
AVM: Arterio-venous malformation
Cr: Creatinine
CT: Computed tomography
eGFR: Estimagted glomerular filtration rate
6F/12F: 6 french/12 french
USS: Ultrasounds scan
